# COVID‐19 information fatigue? A case study of a German university website during two waves of the pandemic

**DOI:** 10.1002/hbe2.260

**Published:** 2021-05-06

**Authors:** Alexander Skulmowski, Bernhard Standl

**Affiliations:** ^1^ Digital Education Institute for Informatics and Digital Education, Karlsruhe University of Education Karlsruhe Germany; ^2^ Informatics Education, Institute for Informatics and Digital Education Karlsruhe University of Education Karlsruhe Germany

**Keywords:** COVID‐19, design, emergency, information, information seeking, page views, pandemic, searches, university, website

## Abstract

The COVID‐19 pandemic has resulted in the ubiquity of health‐related information, disseminated using digital technology. However, recent research suggests that this accessibility of (often negative) information can induce adverse psychological effects, including anxiety, panic‐based hoarding, and other unhealthy behaviors. Some of these consequences have been explained with the idea of an information overload. Considering these current developments, it may become harder to effectively communicate COVID‐19‐related information in smaller, local contexts, such as universities. By analyzing the page views and searches on the website of a university of education in Germany, we derive recommendations for the delivery of information of local organizations. One conclusion is that the need for information during the pandemic decreases as time passes (at least at the local level of institutions such as universities), and even new emergencies such as the beginning of the second wave of COVID‐19 only affect this behavioral pattern to a minor extent. As a result of this COVID‐19 information fatigue, strategies to keep members of institutions informed are discussed. In addition, we suggest developing a mobile app for delivering individualized information right on hand using machine learning and natural language processing strategies. In sum, individual organizations interested in keeping their members informed concerning COVID‐19 should consider the use of personalized information strategies that avoid inducing negative emotional states. Moreover, potentials for connecting people using digital technology could be harnessed in local organizations.

## 1 INTRODUCTION

Although social media and smartphones existed during previous health emergencies such as the 2009 swine flu (H1N1) pandemic, the ongoing COVID‐19 pandemic demonstrates that the lives of people worldwide have become substantially more intertwined with technology use over the last few years. The rapid dissemination of information can be used to keep the international public informed with up‐to‐date knowledge concerning the situation. At the same time, there is a high demand put on the public to keep themselves informed in order to be able to respond to the dynamic nature of this emergency. For instance, the COVID‐19 regulations in many countries set forth that regions can institute different measures based on their respective incidence rates or other criteria. As a result, the public is expected to keep themselves informed concerning both the national and local regulations that are subject to frequent changes, sometimes at short notice. Digital technology plays an unprecedented role in the delivery of this information (see Yan, 2020).

Furthermore, in these difficult times, some of the negative potentials of technology use have became apparent. As can be seen from a social media study conducted using data from the “first wave” of COVID‐19 in 2020, the pandemic has been associated with expressions of loneliness (Koh & Liew, 2020). A survey study suggests that anxiety is related to social media use (Gao et al., 2020; but see also Kaya, 2020). In addition, novel and excessive forms of behavior were observed in the course of the pandemic. These include *cyberchondria*, an extreme form of health‐focused information seeking behavior (Starcevic, Schimmenti, Billieux, & Berle, 2021) as well as the potential for adverse forms of internet use (such as gaming addiction) arising through the lack of social contacts resulting from social distancing (Elhai et al., 2021). Importantly, being exposed to a wealth of information online has been linked to an information overload and cyberchondria (Laato, Islam, Farooq, & Dhir, 2020). One particularly prominent behavioral effect observed was panic buying and hoarding activity (Laato et al., 2020; Naeem, 2021). Furthermore, the possibility for using computer games as an escape from reality has been described (Zhu, 2021). Considering these aspects, the need to stay informed, combined with the vast supply of (at times conflicting) information available using digital technology may lead to significant psychological effects on the population over time.

Due to these actual and potential problems arising through the use of digital technology during the pandemic, the development of effective forms of psychologically safe communication should be supported. In order to handle the pandemic long‐term, anxiety and depression as well as the states of escapism need to be prevented. Thus, (digital) information services concerning the pandemic should focus on delivering relevant facts in a manner that humans can cope with safely over a long period of time. Koh, Chan, and Tan (2020) argue that the ubiquity of COVID‐19 information can induce an *information fatigue*. They summarize that short periods of anxiety invoked through worrying health‐related news can lead to being desensitized after this information has been repeatedly presented (they cite Baseman et al., 2013). This possible effect of COVID‐19 information ubiquity is of high importance and may affect information seeking behavior not only on a global level, but also at a local level, such as when keeping oneself informed of the regulations at one's one workplace, school, or university.

At the occasion of the start of the “second wave” of COVID‐19 in European countries in the Fall of 2020, we were interested to assess how the insights gained from the reviewed literature can be used to improve the information strategy of smaller institutions, an issue that can deliver important pointers for a global communication strategy. In our case study of a university, we were interested to assess whether a COVID‐19 information fatigue had already set in at the local level. In addition, by analyzing web page views of the university, we will present suggestions on how educational institutions can optimally keep their students and staff informed during an ongoing emergency situation that spans several months (and potentially years). In addition to managing digital learning in these times (e.g., Bao 2020; Mahmood, 2021; Skulmowski & Rey, 2020a), we argue that the aspect of information seeking will be an important issue for the education sector. In order to gain insights in people 's information seeking behavior, we discuss the page views and search data of the website of Karlsruhe University of Education (Pädagogische Hochschule Karlsruhe (PHKA), https://www.ph-karlsruhe.de), a German institution of higher education focused on the training of future teachers and educators.

## 2 CASE STUDY

### 2.1 Case context

Karlsruhe University of Education (Karlsruhe, Germany) approximately has 3700 students, 50 professors, 220 academic employees, and 140 non‐academic employees (Manegold, 2020). Thus, it can be considered a smaller, more specialized institution of higher education. The primary function of this university is to prepare teachers for elementary and secondary schools. Additionally, other courses involving education and learning across different ages are offered at PHKA.

### 2.2 Page view data

As most German universities, PHKA created a web page on which the latest news concerning COVID‐19 and its effects on the university are collected (Schneider, 2020). Furthermore, a web page containing advice on how to implement online teaching was introduced at the end of March 2020 and is available for staff only. In addition, staff have access to a web page describing regulations for teaching and exams, and a page outlining the use of the virtual private network (VPN) client used at PHKA.

**FIGURE 1:**
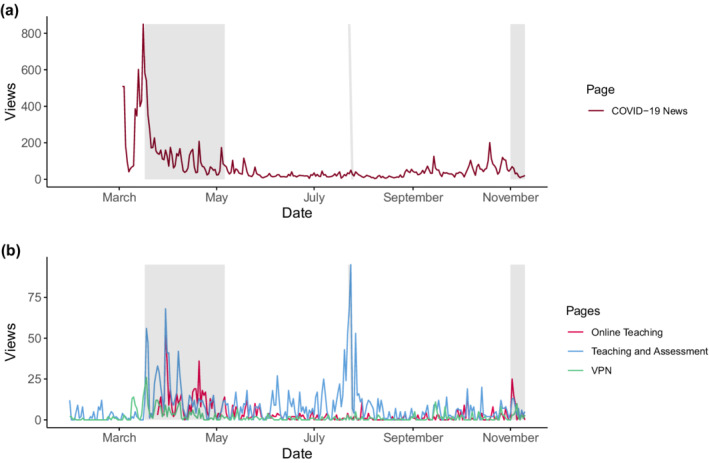
Time series data of page views on web pages of the PHKA website from January 31, 2020 to November 10, 2020. In both panels, the two gray areas roughly signify the two lockdown periods in Germany (the rectangle on the right starts at November 1, 2020, although the partial lockdown officially began on November 2, 2020) while the gray vertical line represents the beginning of the exam period at PHKA at the end of July. (a) Shows the page views of the regularly updated COVID‐19 news page. (b) Depicts the page views of three other pages of the PHKA website: Online teaching resources, regulations concerning teaching and assessment, and the page providing information regarding the setup of a VPN

Figure 1 presents the daily page views of the aforementioned web pages. The time‐series diagram includes major events related to the COVID‐19 pandemic in Germany, such as the start and end of the first “lockdown” period in which the education system and “non‐essential” parts of the economy were shut down accompanied by restrictions concerning social life. This period in March and April 2020 is commonly perceived as the reaction to the “first wave” of COVID‐19 in Germany. Many of the restrictions introduced during the first lockdown were eased or removed during the summer months. As a means of preparing for a “second wave” of COVID‐19 cases in the winter, it was announced on October 28 that a “partial lockdown” would be put in place during the month of November. This second lockdown reinstated several measures put into place during the beginning of the year, such as restrictions for restaurants and hotels, but a number of societal institutions such as schools were not as strongly impacted this time.

As can be seen from Figure 1, the developments just described were a major influence on the website traffic of PHKA. The most obvious effect of the COVID‐19‐related events was the high interest in the COVID‐19 web page during the first wave (see Figure 1(a)). With a peak value of 850 just before the onset of the first lockdown and also right before the start of the summer term in April, the data underline that a timely communication is critical for institution of higher education during emergency situations. Judging from the first peak in page accesses, we can safely assume that the overwhelming majority of PHKA staff and students had a high demand for information concerning the operation of the university during the pandemic. However, in the following months, this interest gradually dwindled and reached its first low during June, with a small increase towards the beginning of the exam period in July. Over time, interest in this page began to return, potentially due a demand concerning information regarding the winter term starting in October. However, the beginning of the actual teaching period was moved to November, coinciding with the second lock‐down.

A striking difference between the first and second lockdown becomes readily apparent from the diagram: The second wave elicited a substantially lower interest in the COVID‐19 news page than the first one. Compared to the average page views of 274.97 for March (starting March 3), this page was viewed on average only 67.9 times per day in October, with no sign of a rising trend in the first days of November. There are a number of plausible explanations for the pattern. While the first lockdown was a major unprecedented change in people's daily lives around the globe, many restrictions became a habit in the course of the year and did not need to be learned anew in the Fall. Thus, a lower demand for information could be a potential consequence. Another explanation would be that a COVID‐19 information fatigue (previously described by Koh et al., 2020) has set in and made the public less interested in news surrounding the issue. Due to the ubiquity of information concerning COVID‐19, the public may have become disinterested of this topic, at least in the sense that no active search for information is perceived to be necessary. In addition, the changes on the campus of PHKA during October have only been minor, such as new regulations concerning at which locations it is required to wear a mask. It needs to be noted that throughout the pandemic, the rectorate of the university regularly sent news bulletins via email to staff and students. Over the course of time, the members of PHKA may have started to rely on these emails rather than checking the news page. In addition, it needs to be noted that staff and students have access to a coronavirus page that compiles information and links to additional web pages addressing specific issues. From September 1 to November 10, this page, however, only received 80 views from employees and 239 views from students. During the height of interest in March, the COVID‐19 news page received a comparable number of views per day rather than in over a month (see Figure 1(a)).

The remaining pages used in this analysis (Figure 1(b)) are part of the internal section of the website of the institution and hence not available to the public. Thus, they have substantially lower page views, but still offer relevant information on how to accommodate information seeking behavior of staff at universities during crisis situations. First, we will discuss the page on online teaching methods launched at the end of March. Between March 25 and April 23, 384 views were counted. Thus, given the number of professors and academic staff, we can assume that the overwhelming majority will have accessed this page at least once during that time. From this, we can derive that resources that help to adapt to a crisis situation are in high demand and should be provided as soon as possible by universities. However, the data also show that during the summer months, interest in this resource also declined, with a small increase towards the end of October and in the first few days of November. Judging from this development, it is clear that providing such resources requires a plan concerning their maintenance and updates. We assume that most academic staff did not need further assistance after familiarizing themselves with the options for online teaching provided by the university. Universities should devise a plan regarding how often such resources are updated and how often academic staff is reminded to check them (e.g., at the beginning of each term).

The next page of interest are the regulations for teaching and assessment. As the restrictions put in place during the first wave significantly limited the possibility to meet in person (in particular to meet as a group), it became necessary to devise alternative methods to conduct classes and exams. Consequently, there was a strong interest in these regulations as indicated by an increase in page views starting at the end of March and continuing throughout April. After a drop in views during the following months, the beginning of the exam period led to increased view counts in July. However, the page did not receive that much attention after this brief period.

Finally, we were interested whether there was a higher demand for the VPN page that describes how a virtual private network can be setup to access network resources provided by PHKA at home. Other than a slight increase in March and April, no major effects of the pandemic on the views of this page could be found.

### 2.3 Search data

In line with a minimalist design idea that avoids a cluttered arrangement, the navigation bar on the PHKA homepage features only five buttons that lead to submenus, namely “University,” “Studying,” “Research,” “Further education,” and “Campus.” In order to speed up the process of finding a particular site, the PHKA homepage features a search function that enables users to search the site. Table 1 lists the five most searched keywords during the time frame from January 30 to November 10. It is noticeable that the search patterns appear to be largely unaffected by the COVID‐19 pandemic. The keywords indicate an interest in the library, the examination office, module handbooks that contain information regarding lectures and classes, the bachelor's thesis, and the school internships that are part of the training of teachers. Searches for the keywords “Corona” and “corona” only amount to 360. Thus, we can assume that the website provides sufficient guidance towards finding relevant and topical information concerning the COVID‐19 pandemic.

**TABLE 1:** Ranking of search keywords and their respective search counts from January 31, 2020 to November 10, 2020

Rank	Keyword	Searches
1	Library	1956
2	Examination office	1750
3	Module handbook	1467
4	ISP (school internship)	1415
5	Bachelor's thesis	1284

*Note*: Keywords using different letter cases were merged, but combinations with different keywords (e.g., “online library”) were not included.

## 3 DISCUSSION

The website data presented in this case study offer insights into the information seeking behavior during the pandemic. In particular, our data set provides a first look on challenges associated with the second wave of COVID‐19. From the page view data, it is possible to draw a number of conclusions. In the beginning of the pandemic, members of the university heavily consulted the COVID‐19 news page, underlining the need for current information on the operation of the university. While there was a tremendous interest in the COVID‐19 news page at the beginning of the pandemic, the second wave did not elicit a comparable need for information. Furthermore, information resources provided by the university were largely only accessed when they were immediately needed. Taken together, it becomes obvious that information is accessed on a need‐to‐know basis, with ongoing developments and changes acting as a catalyst for the need for information. Over time, the demand for information seems to decline and is only weakly affected by new major events related to the pandemic.

In interpreting the data, we would like to avoid an overgeneralization of the limited data set. The dramatic decline in interest in the COVID‐19 news page of PHKA, for instance, should not be considered as evidence for a general disinterest in recent developments concerning the pandemic. However, the data clearly demonstrate a change in information seeking behavior. While the COVID‐19 news page appears to have been a very important place to find information on how the pandemic affects the operation of PHKA during the first wave, no comparable interest could be found in the following months, even during the beginning of the second wave. Although we assume that the members of PHKA kept themselves informed via other media during that time, this development shows that there is less interest in local developments at a particular institution over time.

Koh et al. (2020) described the risk of information fatigue as the result of being exposed to very similar messages and news for an extended period of time. Furthermore, they state that, after a short time span of anxiety, this state can even entail being desensitized to the issue. Further research is needed whether these effects merely lead to a lack of active information seeking, an avoidance of COVID‐19‐related information, or the impression that one does not need additional information on this topic at all. If the latter result was the case, it could become even more difficult to deliver relevant new information to the public.

In any event, this information‐seeking pattern presents a challenge for (university) administrators. How can they be sure that all members of the institution are up to date concerning the newest regulations, even if only minor changes are being made? In the following sections, we will discuss the challenges arising through this pattern and present potential solutions.

### 3.1 Reacting to shifts in information seeking behavior

Based on our data and additional research on the COVID‐19 pandemic and its effect on behavior, institutions of higher education are facing a number of challenges when trying to keep their members up to date on the relevant regulations and restrictions. One particular challenge consists in communicating minor changes such as extensions of the scope of mask regulations. In the example of PHKA, the area on which masks need to be worn was extended to cover the entire campus rather than just the insides of buildings. Instead of assuming that people will deliberately check all new documents and regulations, the most important changes should be communicated in a brief manner (see Chan, Nickson, Rudolph, Lee, & Joynt, 2020). One important insight that can be transferred from research on multimedia learning is that presenting the same information regardless of the prior knowledge of learners leads to detrimental effects on learning (e.g., Kalyuga, 2007; Rey & Buchwald, 2011). This result pattern is referred to as the *expertise reversal effect* (Sweller, Ayres, Kalyuga, & Chandler, 2003). It is assumed that ignoring the level of prior knowledge can lead to various problems during the learning process, such as requiring learners to actively search and compare which part of the presented information is new among all the content they already know or a lowered motivation to engage with material that is deemed as redundant (e.g., Rey & Buchwald, 2011; for an overview, see Schnotz, 2010). When applying this effect to communicating information during a pandemic that spans several months or years, it becomes apparent that reactions to shifts in information seeking behavior may become necessary.

As a general recommendation, COVID‐19 news pages made available by institutions should at all times appear up to date, with no outdated information or other indicators of presenting old, and possibly incorrect, information. Importantly, a more personalized delivery of information could prevent information fatigue. For instance, user accounts could be used to present short push notifications to members of an institution and, by asking for a confirmation, check if the most important changes have been presented to all members. At the same time, the repetitive nature of constant COVID‐19 messages could be avoided.

Furthermore, a change to more incidental communication strategies may be warranted that facilitate the spread of information. For instance, warning signs could be set up on campus to communicate the most important changes. Further technology‐enhanced measures can include digital equipment for communicating measures on site, such as information screens. Such screens can include a near field communication (NFC) interface, which allows members of the university the display of individualized information with NFC chips. Another measure could be the introduction of a mobile university app, which could offer a wide range of possibilities for direct and highly individualized communication of announcements. For a quick access to individualized information, a chatbot could be introduced as part of such an app employing machine learning and natural language processing strategies. Such systems are already available as information applications at universities (e.g., Daswani, Desai, Patel, Vani, & Eirinaki, 2020). A chatbot could not only provide information about the general current guidelines in connection with COVID‐19 but could also be more precisely customized to the user through a more extensive profile.

Moreover, the COVID‐19 emergency situation has been found to affect people in a number of emotional and social ways. A communication strategy will need to accommodate people's emotional states in order to be effective. For instance, the problem of an extreme fear of COVID‐19 has arisen (Starcevic et al., 2021) and can promote forms of unhealthy internet usage (Elhai et al., 2021). Previous research indicates that the COVID‐19 pandemic may lead to an increase in escapism (Zhu, 2021). It will be necessary to monitor this aspect and to prevent students and staff from missing out on important developments. One strategy to counter this development could be to present the information in a manner that avoids an induction of escapism, such as avoiding messages that diminish people's hope regarding an improvement in the future. Given the fact that people are subjected to various forms of dubious or incorrect information concerning the pandemic already (Greenspan & Loftus, 2021), effective communication is a particularly challenging task for leaders of smaller organizations.

In addition to the negative effects described throughout this paper, digital technologies, and social media in particular, have been found to be well‐suited for rapidly disseminating COVID‐19 information to health care workers and medical students (Chan et al., 2020; Huddart et al., 2020). Importantly, successful communication in such contexts hinges on presenting the main information in a straightforward and action‐oriented way (Chan et al., 2020). Furthermore, being socially connected during the emergency situation has been found to contribute towards resilience (Nitschke et al., 2020). Therefore, we argue that digital technology might be used in a healthy way in smaller organizations by providing a social networking functionality, and thereby creating a more direct and local social community (in contrast to large, international, and often anonymous social networks).

On a more general note, the prevention of COVID‐19 information overload and information fatigue is a responsibility of the entire (global) society. As summarized by Koh et al. (2020), the constant delivery of COVID‐19 messages through media outlets may actually have the opposite effect of their intention. They cite the results of Baseman, Revere, and Painter (2013), who found that repeating emergency information through several channels of communication can drastically lower the recall performance for the content of these messages. Furthermore, other studies highlight that even smaller design aspects in communication are of high importance concerning emotional reactions. For instance, a recent study found that even minor changes in the (visual) presentation of pathogens can have a substantial effect. The subjective level of danger felt when being presented with visualizations of pathogens can be affected by their shapes (Skulmowski & Rey, 2020b). As the type of visualization used to accompany information on pathogens can thus potentially contribute to a heightened level of anxiety, the mere selection of graphics that are used in a news report becomes a task with a high responsibility (Skulmowski & Rey, 2020b). Considering the highly emotional responses in the first wave (e.g., Su, Wu, Li, Xue, & Zhu, 2021), the aspect of how and at which rate information is conveyed to the public during such long emergency situations needs to be taken into account. Thus, we recommend that in order to counter information fatigue at a local level, there needs to be a, preferably global, strategy on how to keep the public informed in a healthy and sustainable way. Personalized, incidental, social, and less emotionally draining methods could be an important first step towards this goal.

### 3.2 Conclusion

Our findings provide a first indication of how digital user behavior evolves in such a demanding situation as the COVID‐19 pandemic and how this can be interpreted. Through our analysis of the web page data of a German university, we show that the interest in COVID‐19 related information appears to be in a strong decline compared with the attention generated during the first wave. This pattern puts a strong pressure on the leadership of universities and other local organizations around the globe. The COVID‐19 pandemic is capable of inducing extreme behavioral and emotional reactions, ranging from an excessive interest in health‐related information to escapism. The establishment of more sophisticated methods of communicating COVID‐19 information while avoiding both information overload and information fatigue will be a challenge for the next several months. In particular, personalized messaging using a tone that avoids unhealthy responses such as anxiety and depression might be a strategy to keep people informed over a long period of time in a sustainable way.

## CONFLICT OF INTEREST

The authors declare no conflict of interest.

## Data Availability

The data that support the findings of this study are available from the corresponding author upon reasonable request.
